# Adaptation of the Canine Orthopaedic Index to evaluate chronic elbow osteoarthritis in Swedish dogs

**DOI:** 10.1186/s13028-019-0465-1

**Published:** 2019-06-20

**Authors:** Anna Andersson, Annika Bergström

**Affiliations:** 1 AniCura Animal Hospital Bagarmossen, Ljusnevägen 17, 12848 Bagarmossen, Sweden; 2 Department of Clinical Sciences, Swedish University of Agricultural Sciences, Box 7054, 75007 Uppsala, Sweden

**Keywords:** Canine Orthopaedic Index, Dog, Elbow dysplasia, Osteoarthritis

## Abstract

**Background:**

Owner questionnaires may be used to assess osteoarthritis (OA) in dogs. The validated American College of Veterinary Surgeons’ (ACVS) Canine Orthopaedic Index Questionnaire quantifies quality of life in dogs with orthopaedic disease. This index was modified and translated into Swedish and evaluated for validity, reliability and sensitivity. One group with confirmed moderate elbow dysplasia (n = 117) and one healthy control group (n = 146) without radiographic elbow disease and without lameness were included. Telephone interviews with the dog owners were conducted throughout the study using owner-completed questionnaires.

**Results:**

A 16-item questionnaire developed from an initial data set including 22 items, were able to differentiate between the affected group and the control group with good readability. Validity was measured through factor analysis which yielded a three-factor model accounting for 66.3% of the variance. Cronbach’s α was 0.89 for the total instrument, > 0.7 for stiffness, lameness and function, but < 0.7 for quality of life. Based on the process the modified questionnaire can be used in Swedish, as the ACVS COI, to make intra-patient comparisons and evaluation of disease progression.

**Conclusions:**

A sound owner-completed questionnaire translated into Swedish and modified, able to differ healthy dogs from dogs suffering from chronic osteoarthritis is presented. Performed statistical analysis show the items of the instrument to be reasonable and have high construct validity. The questionnaire may be used in the clinical setting and for research.

**Electronic supplementary material:**

The online version of this article (10.1186/s13028-019-0465-1) contains supplementary material, which is available to authorized users.

## Background

Osteoarthritis (OA) is a degenerative disease commonly seen in aging dogs. OA develops secondary to any joint disease including elbow joint dysplasia (ED) [1, 2]. OA may cause reduced mobility, decreased activity and behavioural changes [3, 4]. Different methods for evaluating OA in dogs exists such as diagnostic imaging, clinical assessment, objective measurements and owner-completed questionnaires.

Several validated owner questionnaires evaluating OA in dogs exist today including the Liverpool Osteoarthritis in Dogs (LOAD), the Canine Orthopaedic Index (COI), the Canine Brief Pain Inventory (CBPI) and the Helsinki Chronic Pain Index (HCPI) [3, 5–10]. The protocols may differ slightly, but mainly they have a similar format [6, 7, 9, 11].

In a study by Walton et al. [4], LOAD; HCPI and CBPI were tested, and construct validity was found between the instruments [4]. In this study weak correlations were also found between the CBPI and peak vertical force symmetry index. The CBPI uses an 11 point numerical rating scale [5], and in the American College of Veterinary Surgeons’ (ACVS) COI this is reduced to 5 rates (from none to extreme). The two instruments are otherwise identical. The fact that the instrument has less alternatives will make it easier to use for telephone interviews. The LOAD questionnaire also have five graded rating scales, with a set up including background lifestyle and mobility [3], while the ACVS COI uses the stiffness, lameness function and quality of life to evaluate OA [12]. In this study we were interested in the ACVS COI, translated into Swedish, for future use including the possibility to easily access lameness, stiffness and function.

The ACVS COI questionnaire is designated to be useful in all dogs with orthopaedic disease disregarding breed, age or gender. A new validation and reliability testing should be performed after translation into a new language to verify that the questionnaire is a good tool to evaluate what it is intended for [13]; in this case OA in dogs.

The methodology for evaluating a translated, already validated questionnaire is performed in several steps. First the questionnaire is tested for readability which includes translation. Any ambiguity or misleading questions (= items) should be clarified. In the next step validity, reliability and sensitivity are tested [3, 4, 13]. At every step, editing of unclear items, or removal of questions deemed to have performed poorly is carried out. After optimizing and removal of items a repeated validity and reliability test is performed.

To ensure face validity, experts read and edit the items as necessary [14, 15].

A factor analysis is used to confirm validity. Factor analysis is used to assess the relationship between different variables. Questions are excluded, if necessary, through identification of common factors.

Construct validity evaluates if the questionnaire measures what it aims to measure, in this case OA severity. Construct validity may be ensured by testing the items on healthy dogs as well as in dogs with chronic OA [13]. This is an important step when the attribute being tested cannot be directly observed. Chronic pain due to OA cannot be seen, but behavioural changes as a result to chronic OA can be observed [6].

Reliability of a questionnaire is important as it ensures that the items measure consistently over time. A standard test of internal consistency is Cronbach’s α [16]. The method evaluates if the items within a group of questions are measuring the same concept, (internal consistency) [17, 18].

All items with a low validity or reliability can be removed. In some cases, it is possible to keep an item despite that, if they are still considered important. If changes are made in the items, a repeated test of factor analysis and Cronbach’s α should be performed to ensure the factor loading pattern [19].

The aim of this study was to test validity and reliability for a Swedish questionnaire based on an already validated questionnaire, the ACVS COI. This Swedish COI is intended to be used both as a web and interview-based questionnaire.

Before the validation process, questions were added for evaluation in addition to the ACVS COI. A few questions included in the HCPI were tested. Also, we aimed to evaluate if a question regarding how often a problem such as lameness was scored differed from how severe the problem was scored by dog owners. In the clinical setting, we find some owners struggling with evaluating how sever a problem is, but they may appear having less problems explaining the occurrence of a problem. For this reason we aimed to evaluate if the two items were comparable or not.

## Methods

### Designing the items

The questionnaire is based on the “ACVS COI”. The ACVS index is developed and validated by Brown [5, 11, 12, 20]. A written consent has been given from ACVS 2016. A few questions from the HCPI [9] were added to the ACVS COI during the development of the Swedish questionnaire. This included questions regarding the dog’s willingness to play and the dog’s vocalization (audible complaining, such as whining or crying).

The ACVS COI includes questions regarding the severity of the lameness. Questions about the frequency of the lameness were added to assess the owner’s interpretation in the initial validation. The questions were translated into Swedish and reformulated to ascertain readability for the intended focus group. The ACVS COI items have five different response options, such as 1 = none, 2 = mild, 3 = moderate, 4 = severe and 5 = extreme. Identical response options were used in the Swedish version. These options also make the items usable for an interview and easy for the owners to grasp. The interviews were conducted by telephone, and the alternatives were spoken, without including the numbered response option, only the text for each item.

Face and content validity were tested by having two experienced Diplomates of the European College of Veterinary Surgeons (ECVS), as well as a statistician expert to review and evaluate the protocol. The language was native Swedish for two of the reviewers and one was bilingual. A first pilot test including 20 dog-owners, showed that some of the questions and response options needed to be edited to better fit the target group.

Emphasis has been put into characterise the clinical symptoms, and how to best describe these in words to allow the owner for easy interpretation of the items.

### Focus group

All veterinarians had an introduction to the questionnaire before starting the interviews and clarifying comments were available as needed in the questionnaires. A total of 344 dog owners were interviewed via telephone by five small animal clinicians. The focus group was randomly selected (computerized) from the Swedish Kennel Club (SKC) register of the breeds American Staffordshire, Bernese Mountain Dog, Labrador retriever, Rottweiler and German Shepherd Dog screened for elbow dysplasia (ED) graded 0 and 2. Dogs with elbow dysplasia were selected because it is a common cause of secondary OA.

The SKC ED screening system is based in the International Elbow Working Group (IEWG) and screening process is performed by specialised radiologists. Breeds at risk to develop ED are radiographically evaluated at an age of 18 months or more in a flexed lateral projection of the elbow. The aim is to register any signs of osteoarthritis [19]. ED0 is defined as a normal joint without any radiological findings of osteoarthritis and in this case constitutes the control group. Dogs with ED1 have mild osteophyte formation < 2 mm, ED2 have moderate osteophyte formation 2–5 mm and ED3 have severe osteophyte formation > 5 mm [19]. ED2 with moderate OA constitutes the diseased group and chosen to decrease any interference with the control group, but without severe OA with a risk to have extreme group analysis.

Individuals randomly chosen were screened for ED between January 2011 and January 2015, at least 2 years prior to the date of the interview.

Before starting the interview regarding QOL, questions intended to ensure ownership and that the dog was still alive, were asked. Interview with the owner, or the handler spending time with the dog was accepted. Only the computerized randomly selected dogs were included in the interview and more dogs, even if available, in the same household were not included.

Furthermore, gender, age, if the dog is intact or not, and hip dysplasia screening scores (according to SKCs register) were noted. For exclusion/inclusion criteria’s questions were asked regarding presence of lameness and veterinary care due to lameness at any point in the dog’s life. The owners were asked to answer all QOL items based on the last month (4 weeks). This differs from the initial ACVS COI were the last 7 days were reported.

### Exclusion criteria

Protocols from deceased dogs or incomplete questionnaires, were excluded. Dogs with lameness appreciated by the owner and/or a veterinarian or those treated for any orthopaedic-related problem were excluded from the control group (ED0). Veterinarians includes any clinical practitioner, not requiring a specialist.

In the ED2 group, individuals with orthopaedic related disease originating from joints other than the elbows were excluded.

### The index standardized score

The number of questions varied amongst the categories of stiffness, lameness, function and quality of life. To ensure equal contribution from each category, a maximum categorical score of one was selected for each category. The standardized score is gained when the sum of raw scores within the category (e.g. function) is divided by the maximum achievable score within the category. The stiffness raw score is 5 to 25 (minimum to maximum) and the standardized score of the each category is 0.2–1 (minimum to maximum).

### Statistical analysis

Descriptive demographic data is presented as total numbers and percentages.

The questions to the owners were ordinal, on a scale from 1 to 5. Therefore, the factor analysis was based on polychoric correlations [21]. The polychoric correlations were then put into the factor procedure to perform a principal axes factor analysis. The factor pattern was rotated using the Varimax criterion. Factor loadings higher than 0.4 indicate that the item is highly correlated with the factor [22]. Communality was added to the factor analysis. The communality describes the item’s variance explained by the factor, and communalities < 0.40 may raise the question if the item belongs to the factor.

To establish any difference between the ED0 and ED2 group (construct validity) a Pearson Chi-Square Test was performed on every item. P-values < 0.05 were considered to have high significance [10].

In order to measure the extent to which the items correlate with each other Cronbach’s α was carried out. Cronbach α values range from 0 to 1.0, it is a coefficient and describes the average inter-correlations among items. Items with loadings > 0.7 were considered to have good reliability [5, 7, 16, 23]. SAS was used for all statistical analysis [SAS Institute Inc. (2014): SAS/Stat User’s Guide. Version 9.4. Cary, N. C., SAS Institute Inc.].

## Results

Demographic data including breed, gender, age, hip dysplasia screening results and if any medical treatment with nonsteroidal anti-inflammatory drug was given are presented in Table 1.

**Table 1:** Demographic data from a population of dogs radiographically evaluated for elbow dysplasia (ED), one control group (ED0) and one osteoarthritic group (ED2)

	ED0 (n = 146)	ED2 (n = 117)
Breed		
American Staffordshire	12 (8.2%)	3 (2.6%)
Bernese Mountain dog	36 (24.6%)	24 (20.5%)
German Shepherd	31 (21.2%)	33 (28.2%)
Labrador Retriever	45 (30%)	33 (28.2%)
Rottweiler	22 (15%)	24 (20.5%)
Gender		
Female	77 (52.7%)	38 (32.5%)
Female spayed	22 (15%)	13 (11.1%)
Male	37 (25.3%)	47 (40.2)
Male castrated	10 (6.8%)	19 (16.2%)
Hip dysplasia scoring		
A	82 (56.25)	50 (42.7%)
B	35 (24%)	38 (32.5%)
C	14 (9.6%)	8 (6.8%)
D	9 (6.2%)	20 (17%)
Not available	6 (6.2%)	1 (0.8%)
Age (years)		
Median	5	5
Mean (SD)	5.2 (1.13)	5.35 (1.12)
NSAID treatment		
Continuous	0	6 (5.1%)
Sometimes	0	27 (23.1%)
No treatment	146 (100%)	84 (71.8%)

Percentage within each ED group is presented

Hip dysplasia screening based on the FCI (Federation Cynologique Internationale) guidelines: A–normal; B—transitional; C—light; D—medium; E—severe

The design included repeated editing of the items (face validity) to develop the initial Swedish questionnaire, based on 22 items divided into the four groups: stiffness, function, gait and QOL. The completion rate for all items took less than 10 min for the dog owners including the background information, which indicates it is easy to use, takes little effort from the respondent and has good readability.

From the 344 interviews, 263 were included in this study after exclusion criteria had been reviewed. The breed distribution was: American Staffordshire (15), Bernese Mountain dog (60), German Shepherd (64), Labrador Retriever (78), and Rottweiler (46).

The healthy, ED group 0 included 146 dogs and the OA, ED 2 group, included 117 dogs.

The 22 items were entered into the orthogonal, varimax-rotated factor analysis. The scree plot suggests that three factors were most significant and accounted for 57.1% of the variance.

To measure if any difference between the ED 0 and ED 2 group could be detected for every separate item a Pearson Chi-Square test was performed.

A significant difference between the two groups (P-values < 0.003) was seen in all the items except for items 4 (difficulty to lay down P = 0.129), 17 (owners perception of the dogs mood *P *= 0.974), 18 (willingness to play P = 0.433), and 20 (owners perception of the dogs QOL P = 0.193). Item 19 (vocalization) had a P-value close to not being significant (0.040).

A final revision was performed to reduce the number of questions. Item 4, 17, 18 and 19 were excluded from the questionnaire. None of these items are included in the ACVS COI. Item 20 (“overall, how would you rate your dog’s quality of life over the last month”) was further analysed since it may be of importance for the overall evaluation and owner perception.

One item was moved from the gait section to the stiffness section. “How often did your dog ‘pay’ for over-activity, with increased pain or stiffness the following day?” The ACVS COI has the item in the gait section, but we included it in the stiffness section. In this step several questions were also removed since they did not add any more information. The initial items included two consecutive questions regarding how often and how severe the lameness was that the owner noticed during walk and trot, respectively. The results from these similar questions were compared and we decided to remove one of them, since the results showed more or less identical answers with only a few percentage discrepancies. To be more comparable to the ACVS COI the severity was retained.

A second factor analysis was performed on the remaining 16 items which identified three new factors accounting for 66.3% of the variance. Factor loadings, indicating a high correlation (> 0.4) for all items except item 20. Factor loadings and communality are shown in Table 2. At this point, item 20 was separated, but included in the analysis.

**Table 2:** Statistical factor analysis, with item loading and communality for a Swedish Canine Orthopaedic Index

Item	Factor	Factor loading	Communality
1. Stiffness in the morning	1	0.67	0.45
2. Stiffness during the day	1	0.82	0.69
3. Ability to rise to standing	1	0.69	0.50
5. General joint discomfort	1	0.80	0.71
6. Ability to jump up	2	0.92	0.88
7. Ability to jump down	2	0.93	0.89
8. Ability to climb up	3	0.97	0.95
9. Ability to climb down	3	0.95	0.92
11. Severity of lameness during mild activity	1	0.72	0.69
13. Severity of lameness during moderate activity	1	0.68	0.62
15. Stiffness the day after moderate activity	1	0.68	0.63
16. Owner awareness of dog’s joint discomfort	1	0.82	0.76
20. QOL during the last month	1	0.37	0.15
21. Decrease in general activity	1	0.61	0.46
22. Concerns about life length	1	0.79	0.63

Cronbach’s α analysis was performed for the total instrument, the four groups (stiffness, function, lameness, and QOL), and for all individual items (item-total correlation) (Table 3). The total instrument as well as all individual items (item-total) indicated they correlated well (> 0.7). Significant values > 0.7 were also measured for the three groups stiffness, function and gait whereas the last biological factor, QOL, loaded to 0.66 suggesting these items correlate less to each other.

**Table 3:** Cronbach’s α for a Swedish Canine Orthopaedic Index

Item	Cronbach’s α
*Total instrument*	*0.89*
*Stiffness group*	*0.84*
1. Stiffness in the morning	0.82
2. Stiffness during the day	0.78
3. Ability to rise to standing	0.82
5. General joint discomfort	0.78
15. Stiffness the day after moderate activity	0.81
*Function group*	*0.78*
6. Ability to jump up	0.73
7. Ability to jump down	0.72
8. Ability to climb up	0.75
9. Ability to climb down	0.71
*Lameness group*	*0.90*
11. Severity of lameness during mild activity	0.86
13. Severity of lameness during moderate activity	0.88
14. Lameness the day after moderate activity	0.86
16. Owner awareness of dog’s joint discomfort	0.87
*QOL (quality of life) group*	*0.66*
21. Decrease in general activity	0.88
22. Concerns about life length	0.88
*Owner perception*	NA
20. QOL during the last month	0.89

Cronbach’s α for the total instrument, for the four groups; stiffness, function, lameness and quality of life (italics text), and for all individual items separately (item-total correlation). Significant values > 0.7. Item-total correlations are the correlations of the individual item with the total scale

The final questionnaire is presented in English and Swedish  (Table 4; Additional file 1). The maximum scoring of the index including the raw score and the standardized score is presented in Table 5.

**Table 4:**
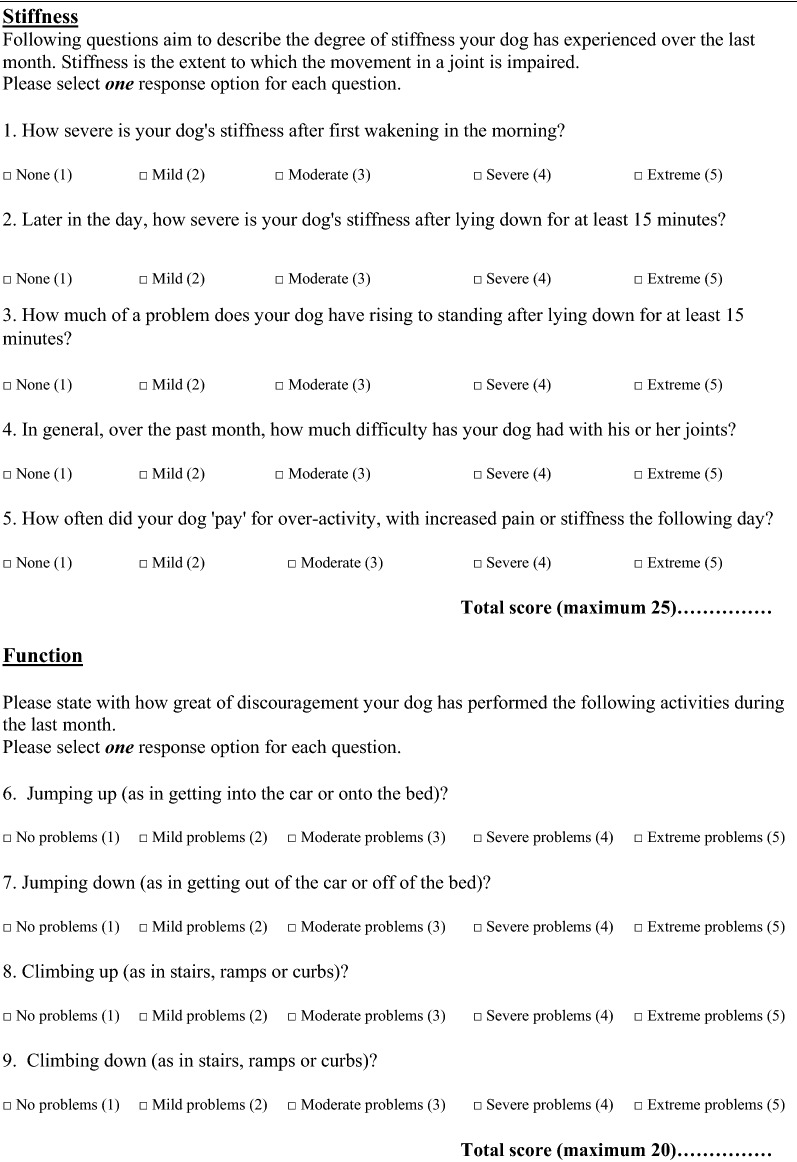

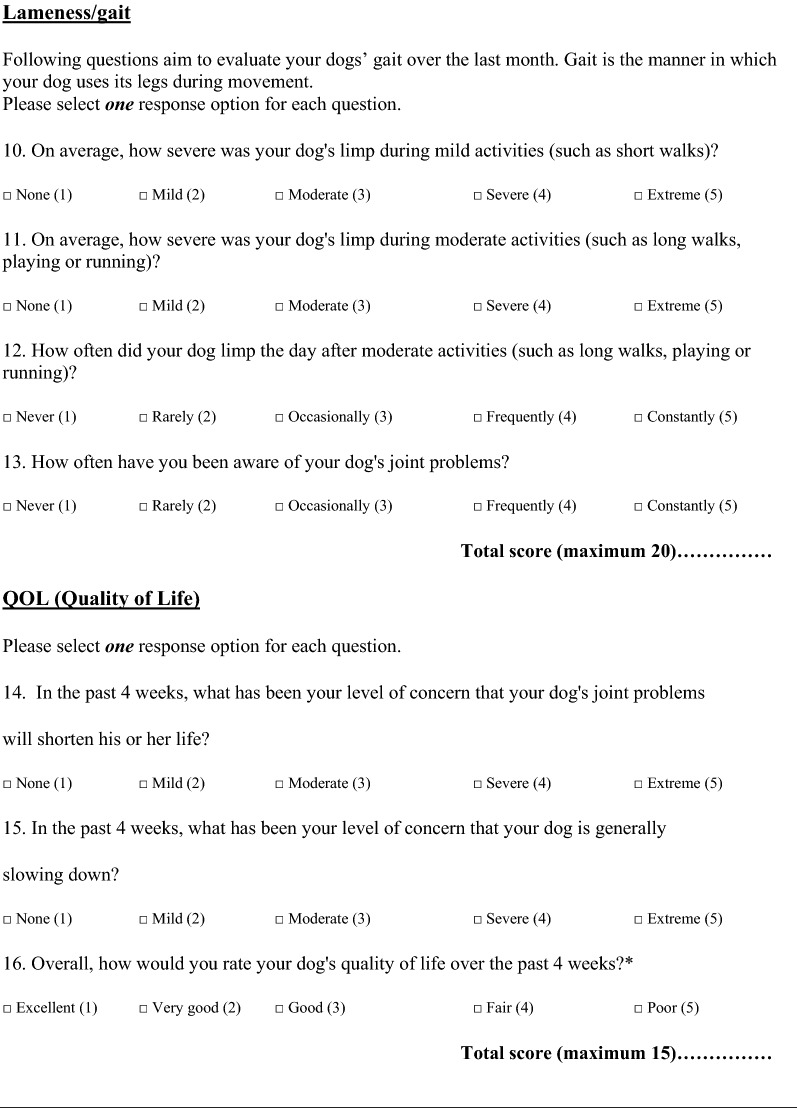
Items included in the final Swedish questionnaire for owner’s interpretation of dogs suffering from chronic osteoarthritis

*Item 16 may be used separately or within the QOL (quality of life) group

**Table 5:** Items 1–16 with maximum raw score and standardized score presented from a Canine Orthopaedic Index

Group/item	Minimum and maximum value (raw score)	Range of the standardized score for each group
Stiffness	5–25	0.2–1
1.	1–5	
2.	1–5	
3.	1–5	
4.	1–5	
5.	1–5	
Function	4–20	0.2–1
6.	1–5	
7.	1–5	
8.	1–5	
9.	1–5	
Lameness/gait	4–20	0.2–1
10.	1–5	
11.	1–5	
12.	1–5	
13.	1–5	
QOL (quality of life)	3–15	0.2–1
14.	1–5	
15.	1–5	
16.	1–5	

## Discussion

Our goal was to develop a reliable owner-completed questionnaire in Swedish for assessing QOL in dogs with orthopaedic disease based on the ACVS COI. We also aimed to make it usable for telephone interviews as well as web and paper-based questionnaires. The access to the large number of screened dogs for ED via the SKK’s register gave us a large target group of 263 dogs and thereby a more reliable validation process. This questionnaire does discriminate dogs with elbow dysplasia from dogs without signs of OA, also the questionnaire has a high internal consistency. The items included in the ACVS COI were all valuable for the task with an exception for one of the QOL items, “the owner’s perception of the dog’s quality of life during the last month”. This question has a very low loading in the factor analysis compared to all other questions in the present study.

The results are consistent with the ACVS COI. A limitation is that only a single type of OA was evaluated. However, based upon the results and the fact that the questionnaire was developed from the validated ACVS COI, it can be applied to other joints. The ACVS COI ha an evaluation period of 1 week and we changed the period to 4 weeks.

The way a questionnaire is formulated and presented to the respondents has been shown to have a significant effect on the result [24]. Although a more extensive questionnaire is considered more credible, it is also more labour-intense on the responding part [25], making the risk of lower completion rate higher. The time to complete the initial 22-item questionnaire was about 10 min. The final, reduced questionnaire includes 16 items and should be even quicker and easier to complete.

The items are based on the ACVS COI, but with some added and one reallocated item. Four of the added questions were removed as they did not differ between the healthy control group and the OA group, and they were not included in the ACVS COI. All four questions could arguably be deleted from the questionnaire. For example the willingness to play or the frequency of vocalisation were more of a personal or breed influence rather than correlated to the amount of pain or decrease in QOL. Our initial question regarding the owner’s perception of severity and frequency was shown not to differ in the questionnaire. Owners answered these items similarly, therefore, the frequency was removed.

From the tests in this study, we found that the item “Overall, how would you rate your dog’s quality of life over the past 4 weeks?” had weak results and there was no significant difference between our healthy control dogs and the OA dogs, a result which differs from the ACVS COI study [5]. We conclude that owner perceived ability to determine QOL do not correlate with lameness and pain. However, this item may still yield important information about the owner’s interpretation of the dog’s QOL. If the item should be included in the index, or stand alone as a separate owner perception, can be discussed.

One observation was that “lameness the day after moderate activity” (item 12) was scored lower compared to “stiffness the day after moderate activity” (item 5) (Table 4). It may be clearer and more visible for an owner to observe pain and stiffness compared to lameness the day after activity. The two items are included in the ACVS COI, and we suggest that both continue to be used, but with the understanding that items may have unequal importance.

We elected to follow the ACVS COI with five written alternatives from normal to severe in our questionnaire, our intention being that this type of questionnaire can be easily used either as a telephone interview, a web-based questionnaire, or a paper-based questionnaire. Adding more alternatives per item may not improve the validity. Other similar questionnaires use 0–10 with 0 being normal and ten being severely bad [6, 9]. This is closer to a VAS scale and therefore a good tool for a web or paper questionnaire, but not for a telephone interview.

The main limitations in this study is the lack of clinical assessment of the animals as well as any objective measurements such as kinetics or kinematics. The dogs included in the study have only been radiographed and scored according to their ED status. ED status is evaluated by a group of very experienced veterinarians at the SKC, making the ED status very reliable. A pronounced significant difference was seen between the healthy control group (ED0) and the OA affected group (ED2), which indicates the questionnaire is sensitive to detect changes in a dog’s behaviour and gait due to orthopaedic disease. It is possible that dogs in the healthy control group may suffer from undiagnosed OA, and we may have dogs in the OA group without significant problems from the radiographically diagnosed OA. However, based on the exclusions criteria, animals with concurrent lameness should have been ruled out. Clinical factors such as body condition score has not been taken into account when evaluating this questionnaire, since veterinary records were not available from this material. Information is based solely on owner information.

The factor analysis revealed a 3-factor model that accounted for 66.3% of the variance comparable to a two-factor model questionnaire, accounting for 76.8% of the variance [7] and a 3-factor model questionnaire accounting for 59.1% of the variance [9], both designed to evaluate pain in dogs with OA. Accordingly, the percentage of variance accounted for by the 3-factor model in this study is within the range of previous designed owner-completed questionnaires for dogs with orthopaedic disease.

The Cronbach’s α analysis results makes it evident that the QOL part of the questionnaire does have some weakness. This was also reported by Brown et al. were the owner’s interpretation of the dogs QOL scored low [11, 12]. From the owner’s standpoint, many factors influence the answer such as if the owner is more anxious and if the dog has other problems, for example, behavioural issues (aggression/fear etc.) or another ongoing disease. It is also likely that in order for an owner to subjectively view the dogs QOL being diminished, changes in several factors influencing the dogs QOL have to be significant affected. It may be more challenging for the owner to estimate the QOL score being “poor” instead of “good” in the dog, compared to evaluating specific factors such as stiffness and lameness which may be more easily assessed. The QOL items are kept as a part of the total instrument but should be valued by its own with circumspection.

No stability test (test retest) has been performed. The test was done in the ACVS COI, our questions as well as results correspond to the results from Brown et al. [11] we concluded this was not needed after our modification and translation.

The modification in the final translated version includes a change in the order of the items (item 13 in the ACVS COI was moved to item 5 in the Swedish version). The owners were asked to response to the questions for the last month, not only the past week. Besides the two, above mentioned changes, the Swedish COI is identical to the ACVS COI.

The introduction of an index with a standardized score may be helpful to equalise the importance of each category stiffness, lameness, function and QOL. In the clinical setting as well as in research it may not be the total sum of the questionnaire, but the different parts that contributes to knowledge regarding the dog or populations of dogs, for example the possibility to evaluate stiffness compared to lameness over time.

The aim of this study was to translate and then re-validate the ACVS COI after translation, the modifications made are in the final version minor and the process answered several questions regarding items that were added and evaluated during the process. We find the ACVS COI to be a well-designed protocol and now a similar version can be used in Swedish.

## Conclusions

We present here an easy to use questionnaire validated and tested for reliability after translation and modification of an already validated protocol for chronic OA (ACVS COI). Performed statistical analysis show the items of the instrument to be reasonable and have high construct validity. The questionnaire may be used for interviews as well as web- or paper-based questionnaires which makes it useful both in the daily based work at the clinic as well as for research or clinical trials (Additional file 1).

## Additional file

**Additional file 1.** The Swedish Canine Orthopeadic Index.

## Data Availability

The background data that support the findings of this study are available from the Swedish Kennel Club but restrictions apply to the availability of these data, which were used under license for the current study. The data from the questionnaires, without identification of individuals are however available from the authors upon reasonable request.

## Abbreviations

ACVS: American College of Veterinary Surgeons
CBPI: Canine Brief Pain Inventory
COI: Canine Orthopaedic Index
ED: elbow joint dysplasia
HCPI: Helsinki Chronic Pain Index
IEWG: International Elbow Working Group
LOAD: Liverpool Osteoarthritis in Dogs
OA: osteoarthritis
SKC: Swedish Kennel Club
QOL: quality of life
