# Supplementary material for: Adaptation of the Canine Orthopaedic Index to evaluate chronic elbow osteoarthritis in Swedish dogs
Source: Acta Vet Scand. 2019 Jun 20;61:29. doi: 10.1186/s13028-019-0465-1 (PMC6585128; doi:10.1186/s13028-019-0465-1)
Supplement: Supplementary file 1 — Additional file 1. The Swedish Canine Orthopeadic Index. [file 13028_2019_465_MOESM1_ESM.docx]

**SWEDISH CANINE ORTHOPAEDIC INDEX**

**Stelhet**

Följande frågor avser beskriva den grad av stelhet din hund har uppvisat under den senaste månaden. Med stelhet menas i hur stor utsträckning rörelsen i en led är nedsatt.

Vänligen välj ***ett*** svarsalternativ för varje fråga.

1. Hur allvarlig bedömer du din hunds stelhet efter att hunden reser sig på morgonen?

□ Ingen (1) □ Lindrig (2) □ Måttlig (3) □ Kraftig (4) □ Mycket kraftig (5)

2. Senare under dagen, hur allvarlig bedömer du din hunds stelhet efter att hunden har legat ner 15 minuter?

□ Ingen (1) □ Lindrig (2) □ Måttlig (3) □ Kraftig (4) □ Mycket kraftig (5)

3. Hur svårt har din hund att resa sig efter att ha legat ner under 15 minuter?

□ Inte alls (1) □ Lindrigt (2) □ Måttligt (3) □ Kraftigt (4) □ Mycket kraftigt (5)

4. Hur svårt bedömer du att din hund haft med sina leder generellt den senaste månaden?

□ Inte alls (1) □ Lindrigt (2) □ Måttligt (3) □ Kraftigt (4) □ Mycket kraftigt (5)

5. Hur ofta får din hund lida för ökad aktivitet med ökad smärta eller stelhet dagen efter?

□ Aldrig (1) □ Vid enstaka tillfälle (2) □ Ibland (3) □ Frekvent (4) □ Konstant (5)

**Total poäng (maximalt 25)………………**

**Funktion**

Ange med hur stor svårighet er hund har utfört nedanstående aktiviteter under den senaste månaden.

Vänligen välj ***ett*** svarsalternativ för varje fråga.

6. Hoppa upp (tex in i bilen, upp i sängen)?

□ Inga problem (1) □ Lindriga problem (2) □ Måttliga problem (3) □ Kraftiga problem (4) □ Mycket kraftiga problem (5)

7. Hoppa ner (tex ut ur bilen, ner från sängen)?

□ Inga problem (1) □ Lindriga problem (2) □ Måttliga problem (3) □ Kraftiga problem (4) □ Mycket kraftiga problem (5)

8. Klättra upp (tex uppför trappa, ramp, trottoar)?

□ Inga problem (1) □ Lindriga problem (2) □ Måttliga problem (3) □ Kraftiga problem (4) □ Mycket kraftiga problem (5)

9.  Klättra ner (tex nedför trappa, ramp, trottoar)?

□ Inga problem (1) □ Lindriga problem (2) □ Måttliga problem (3) □ Kraftiga problem (4) □ Mycket kraftiga problem (5)

**Total poäng (maximalt 20)………………**

**Rörelse**

Följande frågor avser beskriva hundens rörelsemönster under den senaste månaden. Med rörelsemönster menas hur hunden använder sina ben när den går.

Vänligen välj ***ett*** svarsalternativ för varje fråga.

10. Hur allvarlig är din hunds hälta under lättare aktivitet (tex korta promenader)?

□ Ingen (1) □ Lindrig (2) □ Måttlig (3) □ Kraftig (4) □ Mycket kraftig (5)

11. Hur allvarlig är din hunds hälta under måttlig aktivitet (tex långa promenader, lek)?

□ Ingen (1) □ Lindrig (2) □ Måttlig (3) □ Kraftig (4) □ Mycket kraftig (5)

12. Hur ofta haltar din hund efter måttlig aktivitet (tex långa promenader, lek)?

□ Aldrig (1) □ Vid enstaka tillfälle (2 ) □ Ibland (3) □ Frekvent (4) □ Konstant (5)

13. Hur ofta lägger du märke till din hunds ledproblem?

□ Aldrig (1) □ Vid enstaka tillfälle (2) □ Ibland (3) □ Frekvent (4) □ Konstant (5)

**Total poäng (maximalt 20)………………**

**Livskvalitet**

Vänligen välj ***ett*** svarsalternativ för varje fråga.

14. Hur orolig har du varit under de senaste 4 veckorna att din hunds ledproblem ska förkorta hundens liv?

□ Inte alls (1) □ Vid enstaka tillfälle (2) □ Ca en gång/vecka (3)□ Flera gånger i veckan (4) □ Varje dag (5)

15. Hur orolig har du varit de senaste 4 veckorna att din hund generellt saktat ner farten/trappat ned på sina olika aktiviteter?

□ Inte alls (1) □ Vid enstaka tillfälle (2) □ Ca en gång/vecka (3)□ Flera gånger i veckan (4) □ Varje dag (5)

16. Hur upplever du att din hunds generella livskvalitet varit under de senaste 4 veckorna?

□ Utmärkt (1) □ Mycket bra (2) □ Bra (3) □ Dålig (4) □ Kraftigt försämrad (5)

**Total poäng (maximalt 15)………………**
